# Replenishment of Hepatitis B Virus cccDNA Pool Is Restricted by Baseline Expression of Host Restriction Factors In Vitro

**DOI:** 10.3390/microorganisms7110533

**Published:** 2019-11-06

**Authors:** Sergey Brezgin, Anastasiia Kostyusheva, Ekaterina Bayurova, Ilya Gordeychuk, Maria Isaguliants, Irina Goptar, Anastasiia Nikiforova, Valery Smirnov, Elena Volchkova, Dieter Glebe, Dmitry Kostyushev, Vladimir Chulanov

**Affiliations:** 1 National Medical Research Center for Tuberculosis and Infectious Diseases, 127994 Moscow, Russia; ak@rcvh.ru (A.K.); vladimir.chulanov@rcvh.ru (V.C.); 2 Institute of Immunology, Federal Medical Biological Agency, 115522 Moscow, Russia; vall@mail.mipt.ru; 3 NF Gamaleya Research Center of Epidemiology and Microbiology, 123098 Moscow, Russia; 79153645941@ya.ru (E.B.); lab.gord@gmail.com (I.G.); maria.issagouliantis@rsu.lv (M.I.); 4 Chumakov Federal Scientific Center for Research and Development of Immune and Biological Products of Russian Academy of Sciences, 108819 Moscow, Russia; 5 Sechenov First Moscow State Medical University, 119146 Moscow, Russia; az@rcvh.ru; 6 Riga Stradins University, LV-1007 Riga, Latvia; 7 Karolinska Institutet, SE-171 76 Stockholm, Sweden; 8 Izmerov Research Institute of Occupational Health, 105275 Moscow, Russia; probirka@list.ru (I.G.); utkina.anastasia@gmail.com (A.N.); 9 Institute of Medical Virology, University of Giessen, 35392 Giessen, Germany; dieter.glebe@viro.med.uni-giessen.de; 10 Central Research Institute of Epidemiology, 111123 Moscow, Russia

**Keywords:** cccDNA, rcDNA, maintenance, persistence, innate immunity, viral replication, DNA damage, methylation, CRISPR/Cas9, DNMT3A, gene editing

## Abstract

Background: Covalently closed circular DNA (cccDNA) of hepatitis B virus (HBV) is the major cause of viral persistence in patients with chronic HBV infection. Understanding the mechanisms underlying stability and persistence of HBV cccDNA in hepatocytes is critical for developing novel therapeutics and managing chronic hepatitis B. In this study, we observed an unexpected increase in HBV cccDNA levels upon suppression of transcription by de novo DNA methyltransferase DNMT3A and uncovered additional mechanisms potentially involved in HBV cccDNA maintenance. Methods: HBV-expressing cell lines were transfected with a DNMT3A-expressing plasmid. Real-time PCR and HBsAg assays were used to assess the HBV replication rate. Cell cycling was analyzed by fluorescent cell sorting. CRISPR/Cas9 was utilized to abrogate expression of *APOBEC3A* and *APOBEC3B*. Alterations in the expression of target genes were measured by real-time PCR. Results: Similar to previous studies, HBV replication induced *DNMT3A* expression, which in turn, led to reduced HBV transcription but elevated HBV cccDNA levels (4- to 6-fold increase). Increased levels of HBV cccDNA were not related to cell cycling, as DNMT3A accelerated proliferation of infected cells and could not contribute to HBV cccDNA expansion by arresting cells in a quiescent state. At the same time, DNMT3A suppressed transcription of innate immunity factors including cytidine deaminases APOBEC3A and APOBEC3B. CRISPR/Cas9-mediated silencing of *APOBEC3A* and *APOBEC3B* transcription had minor effects on HBV transcription, but significantly increased HBV cccDNA levels, similar to DNMT3A. In an attempt to further analyze the detrimental effects of HBV and DNMT3A on infected cells, we visualized γ-H2AX foci and demonstrated that HBV inflicts and DNMT3A aggravates DNA damage, possibly by downregulating DNA damage response factors. Additionally, suppression of HBV replication by DNMT3A may be related to reduced *ATM/ATR* expression. Conclusion: Formation and maintenance of HBV cccDNA pools may be partially suppressed by the baseline expression of host inhibitory factors including *APOBEC3A* and *APOBEC3B*. HBV inflicts DNA damage both directly and by inducing *DNMT3A* expression.

## 1. Introduction

Chronic hepatitis B is one of the most common chronic infectious diseases in the world, and can ultimately lead to liver cirrhosis and hepatocellular carcinoma (HCC) [1]. After infection, the relaxed-circular DNA (rcDNA) genome of the hepatitis B virus (HBV) is modified and archived in the nucleus of infected hepatocytes as covalently closed circular DNA (cccDNA). This episomal intermediate of HBV replication serves as the template for genomic rcDNA and all HBV proteins forming progeny viruses. HBV cccDNA is a highly stable and persistent form of the viral genome that facilitates HBV persistence in chronically infected patients [2]. Modern antiviral therapies using interferons and/or nucleotide/nucleoside analogs cannot directly target HBV cccDNA and hence fail to eradicate the virus completely [3]. Mechanisms of HBV cccDNA formation and persistence are mostly unknown [4]. Persistence of HBV cccDNA in the nuclei of the infected cells may not just be related to its relative stability, as the half-life of HBV cccDNA in liver cells is rather short according to the most recent estimates [5,6]. Instead, the HBV cccDNA pool is largely maintained by the conversion of rcDNA into cccDNA and (re)infection of susceptible hepatocytes by progeny virions [7]. At the same time, innate immunity may partially account for the relatively low levels of HBV cccDNA observed in cell culture models [8]. Experimentally enforced production of host viral restriction factors like APOBECs can partially deplete HBV cccDNA by directly deaminating and destroying viral genomes [9], or at baseline levels by hypermutating HBV rcDNA, leading to the formation of non-viable HBV genomes [10].

De novo methyltransferase DNMT3A is upregulated during HBV infection [11,12,13]. DNMT3A serves as a host antiviral factor that methylates episomal HBV cccDNA, suppressing its transcription and, thus, viral replication [14]. On the other hand, host DNA is also a target for DNMT3A [15]. Cumulative damage induced by DNMT3A [16] and HBV proteins (HBx [17,18] and HBs [19]) may contribute to the development of HCC. Indeed, increased DNMTs expression is known to be linked to cancer [20].

In this study, we provide novel insights into the effects of DNMT3A on HBV infection and, most importantly, identify innate immunity as an important regulator of HBV cccDNA maintenance.

## 2. Methods

### 2.1. Cell Lines, Cell Culture, and Transfection

The HepG2-1.1meHBV (HepG2-1.1) and HepG2-1.5merHBV (HepG2-1.5) cell lines have been previously described [21,22,23]. Briefly, HepG2-1.1 are human hepatoma HepG2 cells with tet-on inducible 1.1-merHBV (genotype D subtype ayw) transcription from a strong cytomegalovirus (CMV) promoter, while HepG2-1.5 cells produce HBV constitutively from their own HBV wild-type promoters/enhancers. The cells were cultured in Dulbecco’s modified Eagle’s medium supplemented with 10% FBS (Gibco, Thermo Fisher Scientific, Waltham, MA, USA), 1% penicillin/streptomycin, and 1% l-glutamine in 6-well plates. Cells were seeded one day before transfection to reach approximately 70% confluency by the next day, when pcDNA3/Myc-DNMT3A2 (a kind gift from Arthur Riggs (Addgene plasmid #35521)) or vector control were transfected using Lipofectamine3000 (Thermo Fisher Scientific, Waltham, MA, USA; cat. #11668019), according to the manufacturer’s protocol. After 24 h, doxycycline (Sigma Aldrich, St. Louis, MO, USA) (100 ng/mL) was added to HepG2-1.1 cell culture medium for 24 h to induce HBV pre-genome mRNA expression; then, doxycycline-containing medium was discarded, and cells were washed twice with PBS before harvesting for isolation of nucleic acids (day 1 of the study). In parallel, cells were cultured in complete medium without doxycycline for two additional days (day 3 of the study). Results were reproduced in three independent experiments. Alternatively, pcDNA3/Myc-DNMT3A was nucleofected into HepG2-1.1 or HepG2-1.5 cells using Amaxa 4D-Nucleofector^TM^ X Unit (Lonza, Basel, Switzerland) and Lonza Nucleofector (Lonza, Basel, Switzerland), according to the manufacturer’s protocols. In brief, 1 million HepG2-1.1 or HepG2-1.5 cells were nucleofected with 5 μg DNMT3A or 2 μg pMAX-GFP (control), cultured with doxycycline-containing media for 24 h (HepG2-1.1) or without doxycycline (HepG2-1.5), rinsed twice with PBS the next day, and harvested after 48 h. Alternatively, DNMT3A- or control vector-nucleofected cells were incubated for 24 h with 5-azacytidine (5 μM) or DMSO the day after nucleofection, rinsed twice with PBS, and incubated in complete medium without 5-azacytidine for an additional 24 h.

### 2.2. CRISPR/Cas9-Mediated Targeting of APOBEC3s

*APOBEC3A* (*APO3A*) and *APOBEC3B* (*APO3B*) CRISPR interference was performed using the *Streptococcus pyogenes* CRISPR/Cas9 gene editing tool. Target sites were selected in the UCSC genome browser. sgRNAs targeting promoters of *APO3A* and *APO3B* were designed using CCTop sgRNA Design Tool (APOsgRNA) [24]. PCR products encoding sgRNAs under control of the U6 promoter were synthesized as described before using 2-step mutagenic PCR with Q5 High Fidelity Polymerase (New England Biolabs, Ipswich, MA, USA) [21,22]. *APOBEC3s*-targeting CRISPR/Cas9 were nucleofected into HepG2-1.1 or HepG2-1.5 cells, incubated for 72 h, and used for further analyses. A list of the sgRNAs and primers used is presented in Table S1.

### 2.3. Isolation of Nucleic Acids, Reverse Transcription, and PCR Analysis

Nucleic acids were isolated by an AmpliSens Riboprep kit (AmpliSens Biotechnologies, Moscow, Russia) according to the manufacturer’s instructions. Following isolation, each sample was used for three subsequent procedures: (1) RNase-free DNase I enzyme (Thermo Fisher Scientific, Waltham, MA, USA) treatment, re-isolation using AmpliSens Riboprep kit, reverse transcription with AmpliSens Reverta-L (AmpliSens Biotechnologies, Moscow, Russia), and subsequent PCR analysis for S-mRNA (S-RNA), pregenomic RNA (pgRNA), *DNMT3A*, *PKR*, *APO3A*, *APO3B*, *MxA*, *PKR*, *DNA-PKcs*, *RAD51*, *Mre11*, *ATM*, and *ATR* expression using TaqMan probes or SybrGreen (Invitrogen, Thermo Fisher Scientific, Waltham, MA, USA); (2) plasmid-safe ATP-dependent DNase (Epicentre, Illumina Inc., Madison, WI, USA) treatment for 12 h, followed by inactivation of the enzyme at 70 °C for 30 min and semi-quantitative PCR with cccDNA-specific primers, as described previously [25]; and (3) total HBV DNA quantitative analysis using an AmpliSens HBV-monitor-FL kit (AmpliSens Biotechnologies, Moscow, Russia). cccDNA and total HBV DNA levels were normalized to levels of genomic β-globin. Primers are listed in Table S1.

### 2.4. Southern Blot Analysis

HBV cccDNA was isolated by the Hirt procedure and detected by southern blot as described previously [26]. Briefly, the Hirt DNA samples were heated at 85 °C for 5 min to denature rcDNA into single-stranded DNA, followed by plasmid-safe ATP-dependent DNase treatment (Epicentre, Illumina Inc., Madison, WI, USA) at 37 °C for 16 h and inactivation of the enzyme by heating at 70 °C for 30 min. The samples were then separated on 1.2% agarose gel by electrophoresis and blotted onto a HybondTM-N+ membrane (GE Healthcare, Amersham, Buckinghamshire, UK). Biotin-labeled probes were obtained using North2South Biotin Random Prime DNA Labeling kit (Thermo Fisher Scientific, Waltham, MA, USA) according to the manufacturer’s protocol, and used for hybridization. Hybridization was performed with a 30-min pre-hybridization at 55 °C for 30 min in the North2South Chemiluminescent Detection kit hybridization buffer (Thermo Fisher Scientific, Waltham, MA, USA) and subsequent hybridization at 55 °C overnight in hybridization buffer containing 30 ng/mL of the labeled probe. Membranes were washed, blocked, and equilibrated. Probe-target hybrids were visualized using Streptavidin:HRP conjugates and Peroxide/Luminol working solution.

### 2.5. HBsAg Quantification

Conditioned medium from HBV-1.1 cells was harvested, filtered through a 0.2-μm filter (Corning Inc., New York, NY, USA), and used for the Abbott Architect HBsAg assay (Abbott Laboratories, Abbott Park, IL, USA). Results were normalized to cell numbers.

### 2.6. Immunocytochemistry and Fluorescent Microscopy

γ-H2AX foci were detected using immunofluorescence as described previously [27]. Briefly, cells were seeded into each well of a 6-well plate with a glass coverslip. At harvest, cells were fixed in 4% paraformaldehyde for 10 min, washed three times in Tris-HCl (50 mM, pH 8.0), and incubated for 30 min with blocking buffer (0.02% Triton X-100, 10% horse serum, and 150 mM NaCl in Tris-HCl (50 mM, pH 8.0)). Glass coverslips were then incubated with primary rabbit anti-γ-H2AX polyclonal antibodies (ab11174, Acam, Cambridge, UK; 1:1000 dilution in blocking buffer) at room temperature for 1 h, washed three times in washing buffer (0.02% Triton X-100 and 200 mM NaCl in Tris-HCl (50 mM, pH 8.0)), and incubated with secondary Alexa Fluor 488 goat anti-rabbit IgG antibodies (ab150077, Abcam, Cambridge, UK; 1:300 dilution in blocking buffer) and nuclear counterstaining reagent Hoechst33342 (Abcam, Cambridge, UK) at room temperature for 1 h. Coverslips were mounted with Fluoroshield reagent (ab104135, Abcam, Cambridge, UK). Foci were visualized on a Leica DMI6000 microscope with 100× immersion objective. γ-H2AX foci were counted visually or using ImageJ. More than 100 cells in each experimental group were chosen for γH2AX quantitation.

### 2.7. FACS Analysis

For cell cycle analysis, HepG2-1.1 cells were stained with DRAQ5 (Abcam, Cambridge, UK) solution according to the manufacturer’s protocol. DNA content in all experimental conditions was assessed as followed: complete cell culture medium was discarded, and cells were washed twice with 1 × PBS, trypsinized, and carefully resuspended in FBS-containing medium. Detached cells were centrifuged (500× *g* for 5 min), supernatants were discarded, and cell pellets were resuspended in 300 µL of PBS containing 5 µM DRAQ5. Analysis of cell cycle distribution was carried out on a FACScalibur flow cytometer (BD Biosciences, Fraklin Lakes, NJ, USA) with Flowing Software 2.5.1 in the PE-Cy7-A channel; signals were plotted in linear mode. Gates used included G0/G1, S, and G2/M areas. To analyze nucleofection efficiency, HepG2-1.1 cells nucleofected with 2 μg pMAX-GFP were harvested after 48 h as described above and analyzed on a FACScalibur flow cytometer. Percentage of GFP-expressing cells was calculated compared to vector-nucleofected HepG2-1.1 control cells in the FITC-A channel using Flowing Software 2.5.1.

### 2.8. Statistics

Values were expressed as the mean ± standard deviation (SD) of triplicate experiments in GraphPad Prism 7.0 software. Student’s *T*-test or one-way ANOVA, where applicable, with Tukey’s HSD post hoc test were used to compare variables and calculate *p* values to determine statistically significant differences in means.

## 3. Results

### 3.1. Overexpression of DNMT3A Suppresses HBV Replication But Increases cccDNA Levels

Previously, HBV infection and replication have been shown to elevate cellular levels of DNMTs [15]. DNMTs, namely de novo methyltransferase DNMT3A, serve as innate factors that epigenetically silence HBV cccDNA and limit viral transcription and replication [11,12,15]. 

**Figure 1:**
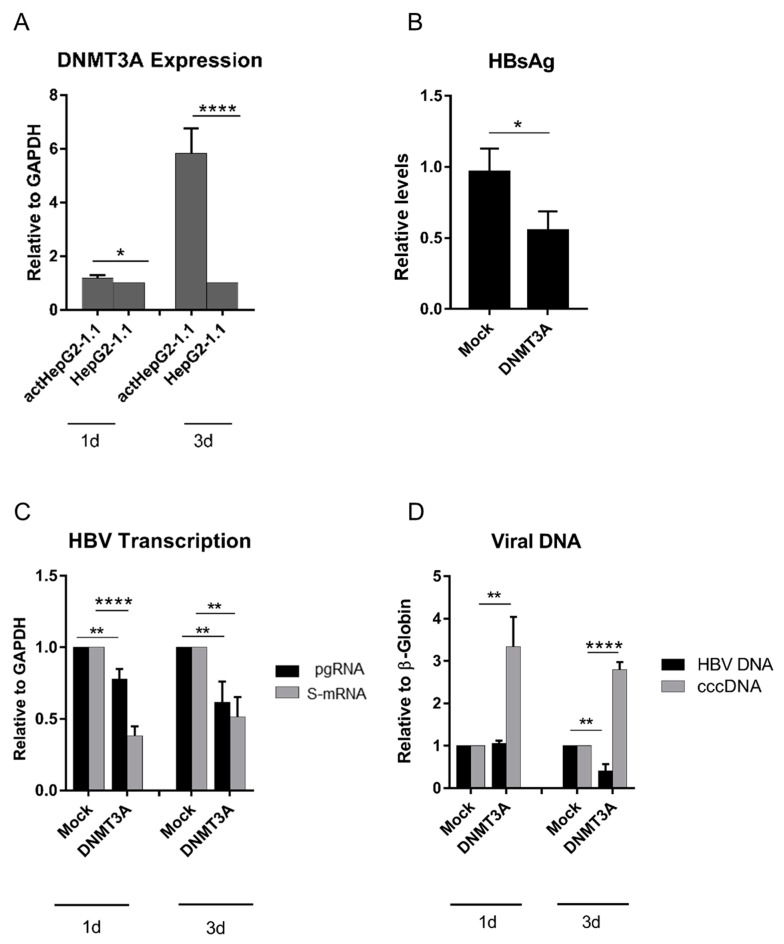
Expression of *DNMT3A* and its effects on HBV replication cycle. (**A**) Activated HBV replication (act) induces expression of *DNMT3A* mRNA in HepG2-1.1. (**B**) Reduction of secreted HBsAg levels on day 3 after *DNMT3A* overexpression in actHepG2-1.1 cells. (**C**) Suppression of HBV genome transcription after *DNMT3A* overexpression in actHepG2-1.1 cells (pgRNA: black bars; S-mRNA: grey bars). (**D**) Decline in total intracellular HBV DNA (black bars) simultaneously with an increase in intracellular HBV cccDNA (grey bars) after *DNMT3A* overexpression in actHepG2-1.1 cells. *DNMT3A* mRNA, pgRNA, and sRNA were measured relative to *GAPDH* mRNA (**A**,**C**), while HBV DNA and cccDNA were normalized to β-globin (**D**). Relative expression levels were calculated using the ΔΔ*C*T method. The values are expressed as means ± SD. Asterisks indicate statistically significant differences. * *p* < 0.05, ** *p* < 0.01, *** *p* < 0.001, **** *p* < 0.0001. Data were analyzed by the *t*-test. *actHepG2-1.1*, cells treated with doxycycline; *HepG2-1.1*, cells not treated with doxycycline; *1d* and *3d*, day 1 and day 3 post transfection or treatment.

Indeed, we detected DNMT3A upon doxyxycline addition, which activates HBV replication in the tet-on inducible HepG2-1.1 cell line, and observed a significant increase in DNMT3A expression (Figure 1A, act-HepG2-1.1, day 3). To precisely investigate whether increased *DNMT3A* expression could interfere with HBV replication, we overexpressed *DNMT3A* in HepG2-1.1 cells (Figure S1), and analyzed its effects on the HBV replication cycle. Surprisingly, secretion of HBsAg and levels of HBV replication intermediates (pgRNA, S-ORF mRNA (S-mRNA)), and total intracellular HBV DNA) were substantially decreased (Figure 1B–D), but the levels of HBV cccDNA were consistently elevated in DNMT3A-overexpressing cells as indicated by PCR (Figure 1D) and southern blot analysis (Figure S2).

**Figure 2:**
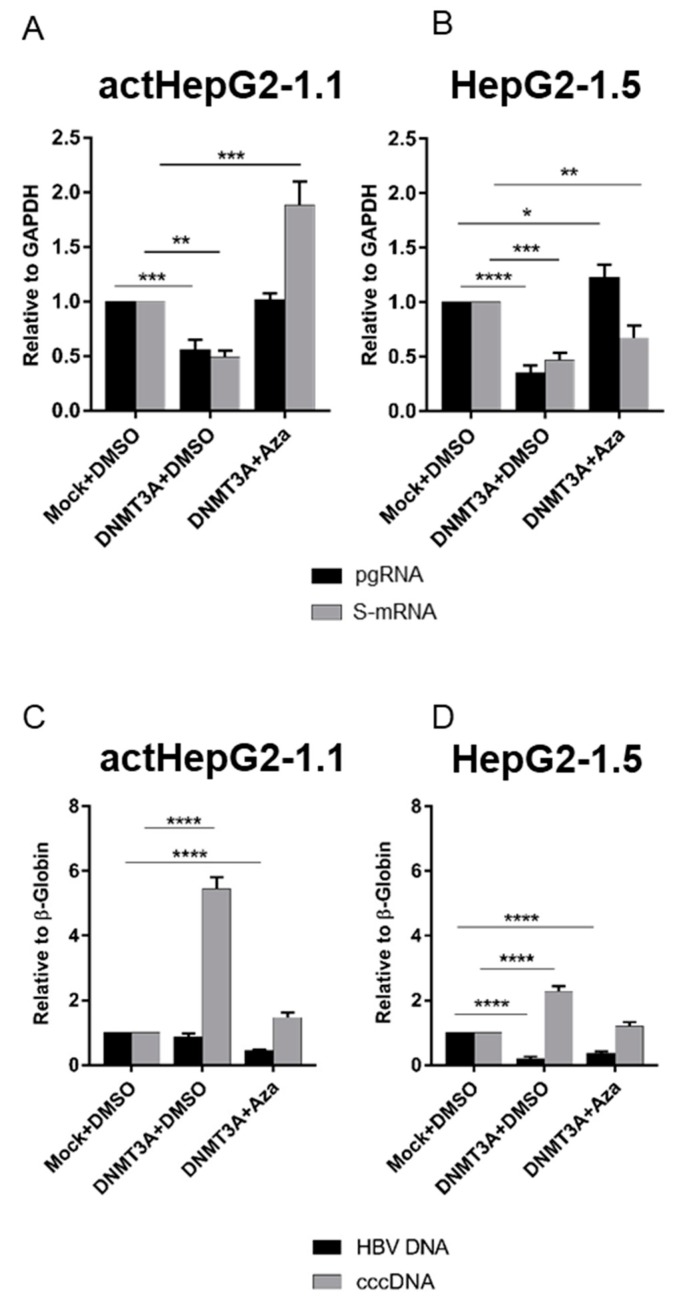
Effects of 5-azacytidine on HBV replication cycle upon *DNMT3A* overexpression. Alterations of (**A**,**B**) HBV transcription (pgRNA: black bars; S-mRNA: grey bars) and (**C**,**D**) total intracellular HBV DNA (black bars) and cccDNA (grey bars) in actHepG2-1.1 and HepG2-1.5 cells. S-mRNA and pgRNA were measured relative to *GAPDH* mRNA, and intracellular HBV DNA and cccDNA relative to β-globin. Relative expression levels were calculated using the ΔΔ*C*T method. The values are expressed as means ± standard deviation. Asterisks indicate statistically significant differences. * *p* < 0.05, ** *p* < 0.01, *** *p* < 0.001, **** *p* < 0.0001. Data were analyzed by one-way ANOVA. *Mock + DMSO*, cells transfected with GFP-expressing plasmid and treated with DMSO; *DNMT3A + DMSO*, cells transfected with DNMT3A-expressing plasmid and treated with DMSO; *DNMT3A + Aza*, cells transfected with DNMT3A-expressing plasmid and treated with 5-azacytidine.

Since previous reports [11,12,15] did not show any significant effects of DNMTs on the HBV cccDNA pool, we investigated whether the observed increase in HBV cccDNA levels was indeed related to increased expression of *DNMT3A*. To test this, we overexpressed *DNMT3A* in the HBV-producing cell lines actHepG2-1.1 and HepG2-1.5 and treated cells with 5-azacytidine (Aza), a strong inhibitor of DNMTs. Treatment with Aza restored viral expression of HBV pgRNA, even when *DNMT3A* was massively overexpressed (Figure 2A,B), and subsequently blocked any increase in HBV cccDNA levels (Figure 2C,D).

### 3.2. DNMT3A Stimulates Cell Cycling in HBV-Infected Cells

Since HBV cccDNA has no origin of replication, the pool of cccDNA becomes diluted when infected cells rapidly divide, and accumulates after infection when cells remain quiescent [5,6,28]. Thus, we hypothesized that overexpressed *DNMT3A* may restrict cell cycling so that quiescent cells accumulate HBV cccDNA [29,30], especially given the observation that conversion of rcDNA to cccDNA occurs predominantly during the G0/G1 phase [28].

**Figure 3:**
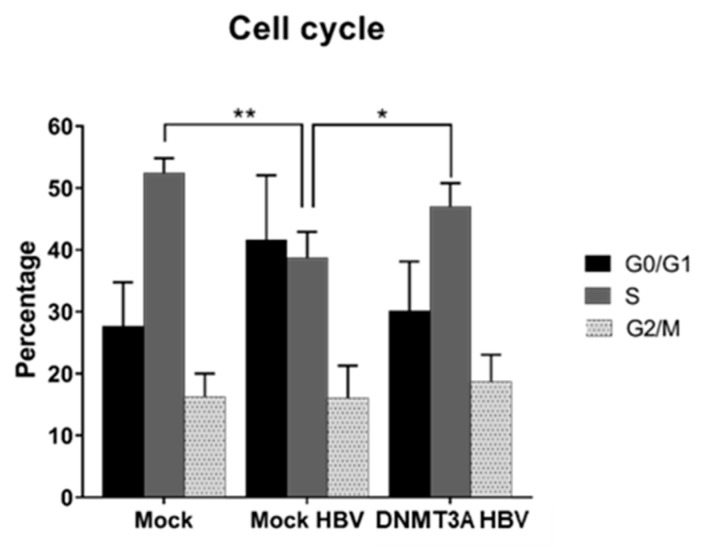
Cell cycling in HBV cell line overexpressing *DNMT3A*. Percentages of HepG2-1.1 cells in different phases of the cell cycle: G0/G1 (black bars), S (grey bars), and G2/M (dotted bars). Asterisks indicate statistically significant differences. * *p* < 0.05, ** *p* < 0.01. Data were analyzed by one-way ANOVA. *Mock*, HepG2-1.1 cells transfected with a GFP-expressing plasmid and without doxycycline; *Mock HBV*, actHepG2-1.1 cells transfected with a GFP-expressing plasmid; *DNMT3A*, actHepG2-1.1 cells transfected with a DNMT3A-expressing plasmid.

As reported earlier, HBV infection is associated with cellular G0/G1 cell cycle arrest [30,31,32], but little is known about the role of DNMT3A expression in this regard. To investigate the effects of elevated *DNMT3A* on cell cycling and HBV replication, we analyzed the cell cycle using FACS. Indeed, HBV replication (mock HBV) resulted in G0/G1 arrest (not significant; *p* = 0.077) and reduced the proportion of cells in the S phase compared to the mock control (*p* < 0.01) (Figure 3). However, transfection of *DNMT3A* strongly stimulated cell cycling, as evidenced by a measurable increase in the proportion of cells in the S phase (*p* < 0.05) together with a decline in G1/G0 cells (not significant; *p* = 0.140) (Figure 3). Overall, DNMT3A appears to stimulate the cell cycle in HBV-producing cells, which leads to a reduction in the number of cells in the G0/G1 phase and increases the cells in the S phase.

### 3.3. DNMT3A Downregulates Expression of HBV Restriction Factors Including APOBEC3s

The HBV cccDNA pool may not be as stable and persistent as previously assumed [7]. A factor for destabilization of HBV cccDNA is the expression of innate restriction factors capable of interfering with HBV replication and cccDNA formation [9,33]. Since DNMT3A was shown to methylate promoters of genes involved in host antiviral defense [34], we investigated the role of DNMT3A expression in the transcription of innate immune system factors during HBV replication.

We analyzed the expression of HBV restriction factors that directly influence HBV cccDNA (i.e., *APOBEC3A* (*APO3A*) and *APOBEC3B* (*APO3B*)), which are directly involved in destroying cccDNA [9,35]. We also analyzed *MxA* [36] and *PKR* [37], which are involved in recognizing HBV and general anti-HBV activity.

**Figure 4:**
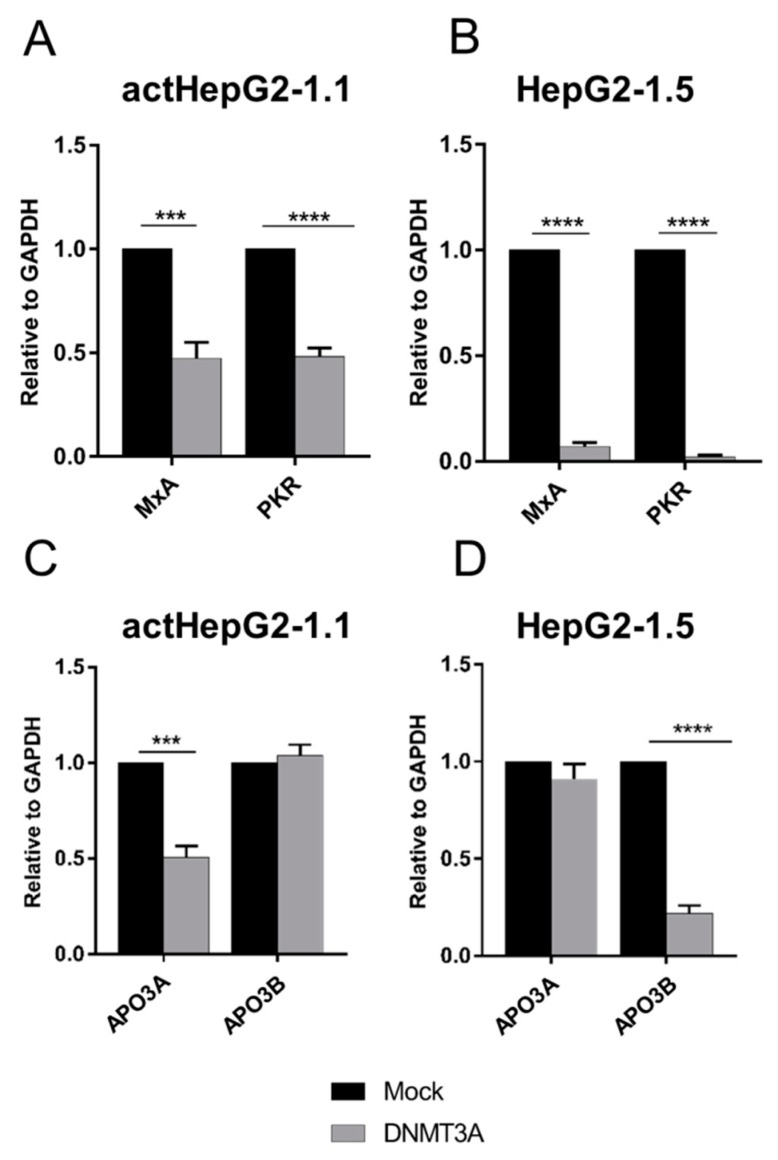
Suppression of host restriction factors by *DNMT3A*. Expression of (**A**,**B**) *MxA* and *PKR*, and (**C**,**D**) *APOBEC3A* (*APO3A*) and *APOBEC3B* (*APO3B*) in HepG2-1.1 and HepG2-1.5 cells. mRNA levels were measured relative to *GAPDH* mRNA. Relative expression levels were calculated using the ΔΔ*C*T method. The values are expressed as means ± standard deviation. Asterisks indicate statistically significant differences. *** *p* < 0.001, **** *p* < 0.0001. Data were analyzed by the *t*-test.

Overexpressing *DNMT3A* in actHepG1-1.1 and HepG2-1.5 cells significantly depressed the transcription of almost all analyzed host restriction factors. Relative levels of *MxA* and *PKR* were suppressed from 52% to 98%, depending on the cell line used (Figure 4A,B). Notably, alterations in APOBECs expression were also cell type-dependent. *APO3A* levels were reduced by half in actHepG2-1.1 cells, but were not altered in HepG2-1.5 cells (Figure 4C,D). In contrast, *APO3B* remained at control levels in actHepG2-1.1 cells, but was dramatically downregulated in HepG2-1.5 cells (Figure 4C,D). As the cell lines differ in HBV replication rates, the observed differences may be attributable to the effects of HBV on the cells.

In conclusion, *DNMT3A* expression downregulates key innate immunity regulators of HBV replication. This may be responsible for the expansion of the HBV cccDNA pool.

### 3.4. CRISPR/Cas9-Mediated Silencing of APO3A or APO3B Increases cccDNA Pool

**Figure 5:**
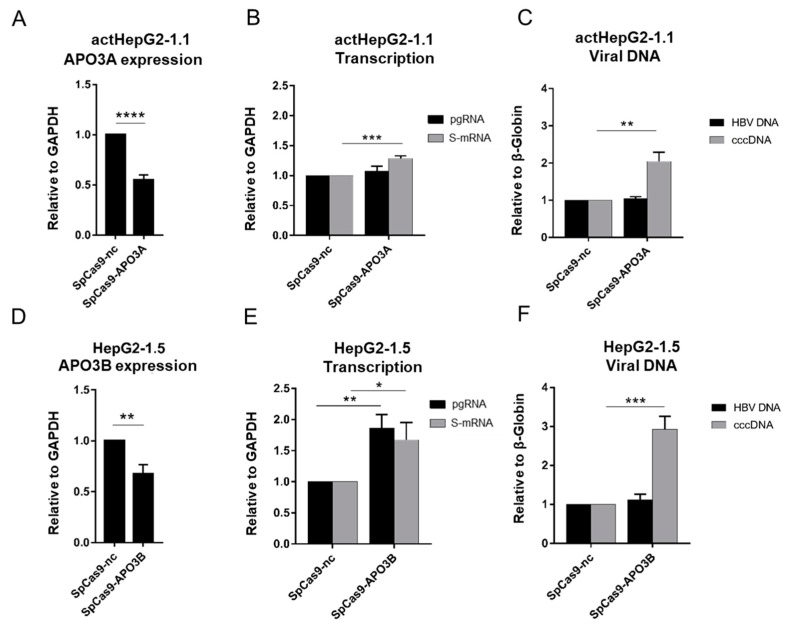
CRISPRi of *APO3A* and *APO3B* genes elevates HBV cccDNA levels. Effects of (**A**) CRISPR/Cas9-mediated *APO3A* silencing on (**B**) HBV transcription (pgRNA: black bars; S-mRNA: grey bars) and (**C**) total intracellular HBV DNA (black bars) and cccDNA (grey bars) levels in actHepG2-1.1 cells. Effects of (**D**) CRISPRi-mediated *APO3B* silencing on (**E**) HBV transcription (pgRNA: black bars; S-mRNA: grey bars) and (**F**) intracellular HBV DNA (black bars) and cccDNA (grey bars) levels in HepG2-1.5 cells. *APO3A*, *APO3B* mRNAs, pgRNA, and S-mRNA were measured relative to *GAPDH* mRNA, and total intracellular HBV DNA and cccDNA relative to β-globin. Relative expression levels were calculated using the ΔΔCT method. The values are expressed as a means ± standard deviation. Asterisks indicate statistically significant differences. * *p* < 0.05, ** *p* < 0.01, *** *p* < 0.001, **** *p* < 0.0001. Data were analyzed by the *t*-test. *SpCas9-nc*, cells transfected with CRISPR system and a non-targeting sgRNA; *SpCas9-APO3A/3B*, cells transfected with CRISPR targeting APO3A or APO3B genes.

APO3A and APO3B are some of the few effector proteins that directly mutate and destroy HBV cccDNA. Lucifora et al. [9] reported that even basal levels of APOBEC3 deaminate cccDNA in different cell culture systems. Likewise, Nair and Zlotnick [10] reported that up to 25% of HBV rcDNA in the supernatant of HepG2-2.15 cells is deaminated by APOBEC3 expressed at baseline levels. To analyze how the baseline levels of APO3A and APO3B affect HBV replication and cccDNA levels, we utilized a CRISPR/Cas9-mediated silencing approach (which disrupts gene promoters by inducing double-stranded breaks in DNA) to shut down the *APO3A* gene in actHepG2-1.1 cells and the *APO3B* gene in HepG2-1.5 cells; these factors were downregulated by DNMT3A expression as previously described (Figure 4C,D). CRISPR/Cas9 system (SpCas9) reduced baseline levels of *APO3A* by 45% in HepG2-1.1 cells (*p* < 0.001, Figure 5A). Silencing *APO3A* using CRISPR/Cas9 in actHepG2-1.1 only slightly altered HBV transcription (Figure 5B), while HBV cccDNA levels increased ~2-fold (Figure 5C), indicating that baseline APO3A does not dramatically affect HBV transcription from HBV cccDNA, but rather impacts the formation and accumulation of cccDNA. In turn, CRISPR/Cas9-mediated disruption of the *APO3B* gene promoter in HepG2-1.5 cells (Figure 5D) decreased APO3B levels, leading to a 2-fold elevation of HBV transcription and ~3-fold increase in HBV cccDNA levels.

Correspondingly, the baseline levels of APO3A and APO3B mildly affected HBV transcription and replication, but significantly limited formation/accumulation of HBV cccDNA.

### 3.5. Overexpressed DNMT3A Reduces Expression of DNA-Damage Response Factors and Aggravates HBV-Related Genome Damage

DNMT3A is one of the many factors involved in HBV-induced HCC development [38]. As reported by us and others, HBV replication itself significantly increases DNA damage, recognizable by the increase of γ-H2AX, a phosphorylated form of histone H2A.X, traditionally regarded as a marker of DNA damage [27,39,40]. One of the key mechanisms of DNMT3A-induced progressive genome instability is the downregulation of genes involved in cell cycle control and DNA repair [41,42].

**Figure 6:**
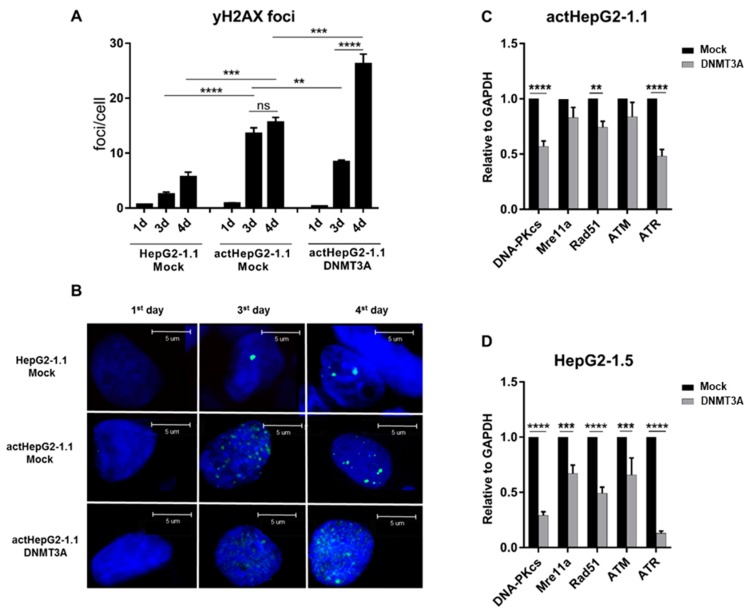
HBV and DNMT3A induce DNA damage. (**A**) Accumulation of γ-H2AX foci in HBV cell lines and *DNMT3A*-overexpressing cells. (**B**) γ-H2AX immunocytochemistry. Cells were stained for γ-H2AX (green); cell nuclei were labeled with Hoechst33342 dye (blue). (**C**,**D**) Alterations in expression of DDR factors in DNMT3A-overexpressing cells. mRNA levels were measured relative to *GAPDH* mRNA. Relative expression levels were calculated using the ΔΔCT method. The values are expressed as means ± standard deviation. Asterisks indicate statistically significant differences. *p < 0.05, **p < 0.01, ***p < 0.001, ****p < 0.0001. Data were analyzed by the *t*-test for γ-H2AX quantitation and one-way ANOVA for gene expression analyses.

In our study, we observed numerous γ-H2AX foci generated in cells with activated HBV replication (Figure 6A,B). Spontaneous γ-H2AX foci were observed in mock HepG2-1.1 cells without doxycycline, but their numbers were significantly lower when compared to cells with active HBV replication (mock actHepG2-1.1). Next, we examined the effects of DNMT3A overexpression on the number of γ-H2AX foci. Overexpression of *DNMT3A* resulted in significantly higher numbers of γ-H2AX foci (Figure 6A,B). Increased formation of γ-H2AX foci in *DNMT3A*-overexpressed cells may be related to aberrant expression of DNA damage repair (DDR) factors. DNA damage repair may be impaired by the methylation of promoter regions in DDR genes by DNMT3A, leading to the downregulation of DDR factor expression, as shown previously [41,42]. To analyze differences in DDR expression in our setting, we measured the expression of DDR factors *DNA-PKcs*, *RAD51*, *MRE11A*, *ATM*, and *ATR* in actHepG2-1.1 and HepG2-1.5 transfected or not transfected with the DNMT3A plasmid. Some of these factors are also involved in HBV replication and reactivation [43].

Constitutive expression of DNMT3A reduced levels of *DNA-PKcs*, *RAD51*, and *ATR* in both cell lines (Figure 6C,D). Notably, DNMT3A may reduce HBV replication not only epigenetically, but also by diminishing *ATM/ATR* levels, as this axis is involved in HBV replication [17,43,44,45].

To conclude, *DNMT3A* expression appears to promote genomic instability by inducing aberrant expression of DDR factors. We also speculate that DNMT3A may limit HBV transcription indirectly by suppressing ATM and ATR intracellular levels.

## 4. Discussion

HBV induces intracellular overexpression of the de novo methyltransferase DNMT3A [15,46]. In a plethora of studies, overexpression of DNMTs was demonstrated to limit HBV transcription and replication [11,15,47]. Surprisingly, in two cell line models, we observed a consistent and reproducible increase in HBV cccDNA levels along with a suppression in HBV transcription when DNMT3A was overexpressed (Figure 1 and Figure 2). We attempted to understand how HBV cccDNA levels can be increased when viral transcription is dramatically reduced. To make sure that the observed effects are related to DNMT3A, we used cells overexpressing DNMT3A and treated with a strong DNMT inhibitor (5-azacytidine) [48] (Figure 2). In these experiments, 5-azacytidine partially restored HBV transcription and alleviated effects of DNMT3A on HBV cccDNA levels (Figure 2).

The most tenable explanation for the increased HBV cccDNA levels in DNMT3A-overexpressing cells seemed to be decelerated cell cycling and, subsequently, reduced dilution and increased accumulation of HBV cccDNA in the cells. However, cell cycle analysis using FACS indicated increased proliferation of cells (Figure 3). Therefore, increased levels of cccDNA upon DNMT3A overexpression are not related to G0/G1 arrest and accumulation of HBV genomes in arrested cells.

Although two cell models used in our study reproduce HBV replication and cccDNA formation, they lack a NTCP receptor and do not support infection and reinfection processes. Additionally, HBV is a slow replicating virus, whereas in HepG2-1.1merHBV (under CMV promoter) and HepG2-1.5merHBV (under wild type HBV promoter) HBV transcription and replication are very active. The viral life cycle is more active in HepG2-1.1merHBV cells when compared to HepG2-1.5merHBV cells, which mimic a more natural infection. In our study, an increase in HBV cccDNA levels upon DNMT3A overexpression was observed in both cell lines. Nevertheless, to better address the biological relevance of the observed phenomenon, more advanced cell models based on transformed (HepG2-NTCP) or primary cell lines (PHH) should be used.

**Figure 7:**
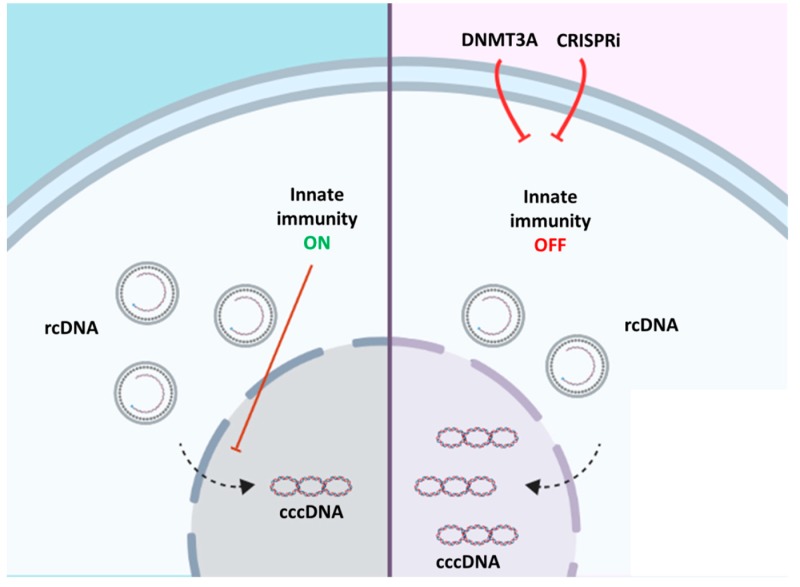
Effects of innate immunity on HBV cccDNA formation and maintenance in in vitro models of HBV replication. Innate immunity limits HBV cccDNA formation at baseline levels (left side; innate immunity ON). Overexpressing *DNMT3A* or CRISPR/Cas9-mediated silencing of factors of innate immunity results in elevated HBV cccDNA levels (right side; innate immunity OFF). The picture was created in BioRender.

Effects of baseline innate immunity factors on HBV cccDNA formation and maintenance were also considered. HBV is a “stealth virus” that does not induce a strong innate immune response [49,50]; for instance, it remains intact upon a strong hepatitis C virus -induced innate immune response [51]. Still, several factors recognize and neutralize HBV cccDNA including APO3A and APO3B [9]. In our studies, DNMT3A demonstrated cell line-dependent effects on APOBECs expression: *APO3A* was predominantly suppressed in actHepG2-1.1 cells, and *APO3B* was downregulated in HepG2-1.5 cells. The reasons for these differences are not clear, but may be indicative of the different innate immune responses and functions in cells with different HBV life cycle activity. Upon doxycycline treatment, HepG2-1.1 cells produce large amounts of viral pgRNA from an artificial, inducible tet-on CMV promoter, and continuously overexpress S-mRNA and HBsAg from HBV-internal promoters, whereas HepG2-1.5 cells produce pgRNA constitutively from a wild-type HBV promoter, mimicking more natural replication of HBV in infected cells. It is well accepted that HBV cccDNA is replenished by conversion from rcDNA to cccDNA, where rcDNA may arise either from rcDNA formed from pgRNA or from de novo infection by rcDNA-containing HBV particles [7]. CRISPR/Cas9-mediated silencing of *APO3A* and *APO3B* had minor effects on viral transcription, but significantly increased HBV cccDNA levels (Figure 5). In previous studies, baseline levels of endogenous APO3A and APO3B had no significant impact on pre-existing HBV cccDNA [10,52]. Instead, a significant proportion of HBV rcDNA was mutated by APOBECs. In this study, we demonstrated that CRISPR/Cas9-mediated silencing of *APO3A* and *APO3B* resulted in significant expansion of the HBV cccDNA pool. One of the possibilities could be that these factors either neutralize a portion of rcDNA, thus reducing the amount of rcDNA able to convert into cccDNA, or directly affect rcDNA to cccDNA conversion (Figure 7). Nevertheless, we cannot exclude that other innate factors in addition to APOBECs or other pathways involved in HBV cccDNA formation could be affected in the conducted experiments.

While we clearly observed that HBV cccDNA levels were increased, and *APOBEC3A/3B* as well as some other factors were downregulated upon *DNMT3A* overexpression, and reduced expression of *APOBEC3A/3B* resulted in an increase in HBV cccDNA comparable to that observed upon DNMT3A overexpression, we cannot exclude that *APOBEC3A/3B* are not the only or the major factors responsible for the observed increase in HBV cccDNA levels when viral transcription is suppressed. We hypothesize that factors of innate immunity or additional host factors may restrict HBV cccDNA formation by limiting conversion of HBV rcDNA into cccDNA or by other mechanisms involving different steps of HBV cccDNA formation. Alternatively, Li et al. [53] observed a modest decline in HBV cccDNA counts using a FISH technique upon TDF treatment (a strong inhibitor reverse transcription) in the early days of the experiment, followed by a drop in HBV cccDNA levels and hypothesized that this slow initial decline may be related to the conversion of mature, residual rcDNA to cccDNA. In particular, Li et al. hypothesized that cccDNA amplification upon TDF treatment may depend on the host kinases and phosphatases responsible for the modification of viral proteins and enhanced transport of core particles into the nucleus [54]. The latter is not likely the case in this study because DNMT3A generally suppresses transcription of the host factors including those potentially involved in cccDNA formation/amplification. Most likely, the observed increase in HBV cccDNA is related to the inhibition of the host factors that restrict cccDNA formation at baseline levels.

Elucidating the mechanisms of the observed phenomenon will be important in understanding the fundamental mechanisms of HBV-cell host interaction, which might provide fundamental insights into HBV biology, or potentially identify novel therapeutic targets for HBV treatment.

Additionally, we asked how HBV and overexpressed *DNMT3A* alter the genome stability of the cell. It is well known that DNMT3A can induce hypermethylation of the host cell genome, alter endogenous expression of DDR factors, and thus contribute to genome stability [15,55]. HBV itself appears to inflict DNA damage, as evidenced by increased formation of γ-H2AX foci, while overexpressed *DNMT3A* increases DNA damage. Generation of γ-H2AX foci in cells with active HBV life cycle was mainly attributable to virologic effects; overexpressing *DNMT3A* suppressed HBV replication, but resulted in more severe cellular genome damage. We speculate that it might occur due to aberrant expression of DDR factors, as described in our study.

To conclude, our study demonstrates for the first time that factors of innate immunity may strongly limit the formation of HBV cccDNA, at least in in vitro models of HBV replication.

## Supplementary Materials

Supplementary materials can be found at https://www.mdpi.com/2076-2607/7/11/533/s1.

## References

[1] Ganem D., Prince A.M. (2004). Hepatitis B virus infection—Natural history and clinical consequences. N. Engl. J. Med..

[2] Nassal M. (2015). HBV cccDNA: Viral persistence reservoir and key obstacle for a cure of chronic hepatitis B. Gut.

[3] Yang H.-C., Kao J.-H. (2014). Persistence of hepatitis B virus covalently closed circular DNA in hepatocytes: molecular mechanisms and clinical significance. Emerg. Microbes Infect..

[4] Schreiner S., Nassal M. (2017). A Role for the Host DNA Damage Response in Hepatitis B Virus cccDNA Formation—And Beyond?. Viruses.

[5] Lutgehetmann M., Volz T., Koepke A., Broja T., Tigges E., Lohse A.W., Fuchs E., Murray J.M., Petersen J., Dandri M. (2010). In Vivo Proliferation of Hepadnavirus-Infected Hepatocytes Induces Loss of Covalently Closed Circular DNA in Mice. Hepatology.

[6] Allweiss L., Volz T., Giersch K., Kah J., Raffa G., Petersen J., Lohse A.W., Beninati C., Pollicino T., Urban S. (2018). Proliferation of primary human hepatocytes and prevention of hepatitis B virus reinfection efficiently deplete nuclear cccDNA in vivo. Gut.

[7] Ko C., Chakraborty A., Chou W.-M., Hasreiter J., Wettengel J.M., Stadler D., Bester R., Asen T., Zhang K., Wisskirchen K. (2018). Hepatitis B virus (HBV) genome recycling and de novo secondary infection events maintain stable cccDNA levels. J. Hepatol..

[8] Busca A., Kumar A. (2014). Innate immune responses in hepatitis B virus (HBV) infection. Virol. J..

[9] Lucifora J., Xia Y., Reisinger F., Zhang K., Stadler D., Cheng X., Sprinzl M.F., Koppensteiner H., Makowska Z., Volz T. (2014). Specific and nonhepatotoxic degradation of nuclear hepatitis B virus cccDNA. Science.

[10] Nair S., Zlotnick A. (2018). Asymmetric Modification of Hepatitis B Virus (HBV) Genomes by an Endogenous Cytidine Deaminase inside HBV Cores Informs a Model of Reverse Transcription. J. Virol..

[11] Vivekanandan P., Thomas D., Torbenson M. (2009). Methylation regulates hepatitis B viral protein expression. J. Infect. Dis..

[12] Guo Y., Li Y., Mu S., Zhang J., Yan Z. (2009). Evidence that methylation of hepatitis B virus covalently closed circular DNA in liver tissues of patients with chronic hepatitis B modulates HBV replication. J. Med. Virol..

[13] Zhang Y., Mao R., Yan R., Cai D., Zhang Y., Zhu H., Kang Y., Liu H., Wang J., Qin Y. (2014). Transcription of hepatitis B virus covalently closed circular DNA is regulated by CpG methylation during chronic infection. PLoS ONE.

[14] Kostyushev D.S., Zueva A.P., Brezgin S.A., Lipatnikov A.D., Simirskii V.N., Glebe D., Volchkova E.V., Shipulin G.A., Chulanov V.P. (2017). Overexpression of DNA-methyltransferases in persistency of cccDNA pool in chronic hepatitis B. Ter. Arkhiv.

[15] Vivekanandan P., Daniel H.D.-J., Kannangai R., Martinez-Murillo F., Torbenson M. (2010). Hepatitis B virus replication induces methylation of both host and viral DNA. J. Virol..

[16] Jin B., Robertson K.D. (2013). DNA methyltransferases, DNA damage repair, and cancer. Adv. Exp. Med. Biol..

[17] Kim S., Lee H.-S., Ji J.-H., Cho M.-Y., Yoo Y.-S., Park Y.-Y., Cha H.-J., Lee Y., Kim Y., Cho H. (2015). Hepatitis B virus X protein activates the ATM-Chk2 pathway and delays cell cycle progression. J. Gen. Virol..

[18] Becker S.A., Lee T.-H., Butel J.S., Slagle B.L. (1998). Hepatitis B virus X protein interferes with cellular DNA repair. J. Virol..

[19] Hsieh Y.-H., Su I.-J., Wang H.-C., Chang W.-W., Lei H.-Y., Lai M.-D., Chang W.-T., Huang W. (2004). Pre-S mutant surface antigens in chronic hepatitis B virus infection induce oxidative stress and DNA damage. Carcinogenesis.

[20] Tarocchi M., Polvani S., Marroncini G., Galli A. (2014). Molecular mechanism of hepatitis B virus-induced hepatocarcinogenesis. World J. Gastroenterol..

[21] Kostyushev D., Brezgin S., Kostyusheva A., Zarifyan D., Goptar I., Chulanov V. (2019). Orthologous CRISPR/Cas9 systems for specific and efficient degradation of covalently closed circular DNA of hepatitis B virus. Cell. Mol. Life Sci..

[22] Kostyushev D., Kostyusheva A., Brezgin S., Zarifyan D., Utkina A., Goptar I., Chulanov V. (2019). Suppressing the NHEJ pathway by DNA-PKcs inhibitor NU7026 prevents degradation of HBV cccDNA cleaved by CRISPR/Cas9. Sci. Rep..

[23] Kostyusheva A.P., Kostyushev D.S., Brezgin S.A., Zarifyan D.N., Volchkova E.V., Chulanov V.P. (2019). Small Molecular Inhibitors of DNA Double Strand Break Repair Pathways Increase the ANTI-HBV Activity of CRISPR/Cas9. Mol. Biol..

[24] Stemmer M., Thumberger T., del Sol Keyer M., Wittbrodt J., Mateo J.L. (2015). CCTop: An intuitive, flexible and reliable CRISPR/Cas9 target prediction tool. PLoS ONE.

[25] Gao Y.-T., Han T., Li Y., Yang B., Wang Y.-J., Wang F., Jing X., Du Z. (2010). Enhanced specificity of real-time PCR for measurement of hepatitis B virus cccDNA using restriction endonuclease and plasmid-safe ATP-dependent DNase and selective primers. J. Virol. Methods.

[26] Cai D., Nie H., Yan R., Guo J.-T., Block T.M., Guo H. (2013). A southern blot assay for detection of hepatitis B virus covalently closed circular DNA from cell cultures. Methods Mol. Biol..

[27] Kostyushev D.S., Brezgin S.A., Kostyusheva A.P., Lipatnikov A.D., Simirskii V.N., Mamonova N.A., Volchkova E.V., Maleyev V.V., Chulanov V. (2018). Increased formation of phosphorylated H2AX foci in nuclei of cells infected by hepatitis B AND B+D viruses. Vopr. Virusol..

[28] Chong C.-L., Chen M.-L., Wu Y.-C., Tsai K.-N., Huang C.-C., Hu C.-P., Jeng K.-S., Chou Y.-C., Chang C. (2011). Dynamics of HBV cccDNA expression and transcription in different cell growth phase. J. Biomed. Sci..

[29] Guo H., Mao R., Block T.M., Guo J.-T. (2010). Production and Function of the Cytoplasmic Deproteinized Relaxed Circular DNA of Hepadnaviruses. J. Virol..

[30] Yeh C.T., Chiu H.T., Chu C.M., Liaw Y.F. (1998). G(1) phase dependent nuclear localization of relaxed-circular hepatitis B virus DNA and aphidicolin-induced accumulation of covalently closed circular DNA. J. Med. Virol..

[31] Sirma H., Giannini C., Poussin K., Paterlini P., Kremsdorf D., Brechot C. (1999). Hepatitis B virus X mutants, present in hepatocellular carcinoma tissue abrogate both the antiproliferative and transactivation effects of HBx. Oncogene.

[32] Gearhart T.L., Bouchard M.J. (2010). Replication of the hepatitis B virus requires a calcium-dependent HBx-induced G1 phase arrest of hepatocytes. Virology.

[33] Qiao Y., Han X., Guan G., Wu N., Sun J., Pak V., Liang G. (2016). TGF-beta triggers HBV cccDNA degradation through AID-dependent deamination. FEBS Lett..

[34] Zheng D.-L., Zhang L., Cheng N., Xu X., Deng Q., Teng X.-M., Wang K.-S., Zhang X., Huang J., Han Z.-G. (2009). Epigenetic modification induced by hepatitis B virus X protein via interaction with de novo DNA methyltransferase DNMT3A. J. Hepatol..

[35] Xia Y., Stadler D., Lucifora J., Reisinger F., Webb D., Hoesel M., Michler T., Wisskirchen K., Cheng X., Zhang K. (2016). Interferon-gamma and Tumor Necrosis Factor-alpha Produced by T Cells Reduce the HBV Persistence Form, cccDNA, Without Cytolysis. Gastroenterology.

[36] Gordien E., Rosmorduc O., Peltekian C., Garreau F., Bréchot C., Kremsdorf D. (2001). Inhibition of hepatitis B virus replication by the interferon-inducible MxA protein. J. Virol..

[37] Park I.-H., Baek K.-W., Cho E.-Y., Ahn B.-Y. (2011). PKR-dependent mechanisms of interferon-α for inhibiting hepatitis B virus replication. Mol. Cells.

[38] Oh B.-K., Kim H., Park H.-J., Shim Y.-H., Choi J., Park C., Park Y.N. (2007). DNA methyltransferase expression and DNA methylation in human hepatocellular carcinoma and their clinicopathological correlation. Int. J. Mol. Med..

[39] Ren J.-H., Chen X., Zhou L., Tao N.-N., Zhou H.-Z., Liu B., Li W.-Y., Huang A.-L., Chen J. (2016). Protective Role of Sirtuin3 (SIRT3) in Oxidative Stress Mediated by Hepatitis B Virus X Protein Expression. PLoS ONE.

[40] Matsuda Y., Wakai T., Kubota M., Osawa M., Takamura M., Yamagiwa S., Aoyagi Y., Sanpei A., Fujimaki S. (2013). DNA Damage Sensor γ-H2AX Is Increased in Preneoplastic Lesions of Hepatocellular Carcinoma. Sci. World J..

[41] Teodoridis J.M., Strathdee G., Brown R. (2004). Epigenetic silencing mediated by CpG island methylation: potential as a therapeutic target and as a biomarker. Drug Resist. Updates.

[42] Ren J., Chu Y., Ma H., Zhang Y., Zhang X., Zhao D., Li Z., Wang J., Gao Y., Xiao L. (2014). Epigenetic Interventions Increase the Radiation Sensitivity of Cancer Cells. Curr. Pharm. Des..

[43] Zhao F., Hou N.-B., Song T., He X., Zheng Z.-R., Ma Q.-J., Li L., Zhang Y.-H., Zhong H. (2008). Cellular DNA repair cofactors affecting hepatitis B virus infection and replication. World J. Gastroenterol..

[44] Kostyusheva A., Brezgin S., Bayurova E., Gordeychuk I., Isaguliants M., Goptar I., Urusov F., Nikiforova A., Volchkova E., Kostyushev D. (2019). ATM and ATR Expression Potentiates HBV Replication and Contributes to Reactivation of HBV Infection upon DNA Damage. Viruses.

[45] Matsuda Y., Ichida T. (2009). Impact of hepatitis B virus X protein on the DNA damage response during hepatocarcinogenesis. Med. Mol. Morphol..

[46] Park I.Y., Sohn B.H., Yu E., Suh D.J., Chung Y.H., Lee J.H., Surzycki S.J., Lee Y.I. (2007). Aberrant Epigenetic Modifications in Hepatocarcinogenesis Induced by Hepatitis B Virus X Protein. Gastroenterology.

[47] Vivekanandan P., Thomas D., Torbenson M. (2008). Hepatitis B viral DNA is methylated in liver tissues. J. Viral Hepat..

[48] Christman J.K. (2002). 5-Azacytidine and 5-aza-2′-deoxycytidine as inhibitors of DNA methylation: Mechanistic studies and their implications for cancer therapy. Oncogene.

[49] Cheng X., Xia Y., Serti E., Block P.D., Chung M., Chayama K., Rehermann B., Liang T.J. (2017). Hepatitis B virus evades innate immunity of hepatocytes but activates cytokine production by macrophages. Hepatology.

[50] Liu H.-Y., Zhang X.-Y. (2015). Innate immune recognition of hepatitis B virus. World J. Hepatol..

[51] Mutz P., Metz P., Lempp F.A., Bender S., Qu B., Schöneweis K., Seitz S., Tu T., Restuccia A., Frankish J. (2018). HBV bypasses the innate immune response and does not protect HCV from antiviral activity of interferon. Gastroenterology.

[52] Seeger C., Sohn J.A. (2016). Complete spectrum of CRISPR/Cas9-induced mutations on HBV cccDNA. Mol. Ther..

[53] Li M., Sohn J.A., Seeger C. (2018). Distribution of Hepatitis B Virus Nuclear DNA. J. Virol..

[54] Perlman D.H., Berg E.A., O’connor P.B., Costello C.E., Hu J. (2005). Reverse transcription-associated dephosphorylation of hepadnavirus nucleocapsids. Proc. Natl. Acad. Sci. USA.

[55] Kuss-Duerkop S.K., Westrich J.A., Pyeon D. (2018). DNA Tumor Virus Regulation of Host DNA Methylation and Its Implications for Immune Evasion and Oncogenesis. Viruses.

