# Supplementary material for: Replenishment of Hepatitis B Virus cccDNA Pool Is Restricted by Baseline Expression of Host Restriction Factors In Vitro
Source: Microorganisms. 2019 Nov 6;7(11):533. doi: 10.3390/microorganisms7110533 (PMC6920784; doi:10.3390/microorganisms7110533)
Supplement: Supplementary file 1 [file microorganisms-07-00533-s001.pdf]

Supplementary

Replenishment of Hepatitis B Virus cccDNA Pool Is Restricted by Baseline Expression of Host Restriction Factors In Vitro

Sergey Brezgin *, Anastasiia Kostyusheva, Ekaterina Bayurova, Ilya Gordeychuk, Maria Isaguliants, Irina Goptar, Anastasiia Nikiforova, Valery Smirnov, Elena Volchkova, Dieter Glebe, Dmitry Kostyushev * and Vladimir Chulanov

Table S1. Primers and probes used in the study.

No	Name	Sequence	Explanation
1	GAPDH_f	CCAGGTGGTCTCCTCTGACTT	qRT-PCR reference housekeeping gene
2	GAPDH_r	GTTGCTGTAGCCAAATTCGTTGT	
3	Gapdh_probe	FAM-AACAGCGACACCCACTCCTCCACC-BHQ1	
4	pgrna_f	GGTCCCCTAGAAGAAGAACTCCCT	qRT-PCR HBV pgRNA transcription
5	pgrna_r	CATTGAGATTCCCGAGATTGAGAT	
6	pgrna_probe	FAM-TCTCAATCGCCGCGTCGCAGA-BHQ1	
7	srna_f	TCCTCCAACTTGTCCTGGTTATC	qRT-PCR HBV S-mRNA transcription
8	srna_r	AGATGAGGCATAGCAGCAGGAT	
9	srna_probe	FAM-ATGATAAAACGCCGCAGACACATCCAGC-BHQ1	
10	HBV_DNA_f	Available in AmpliSens® HBV-Monitor-FL kit	qRT-PCR total HBV DNA level
11	HBV_DNA_r	Available in AmpliSens® HBV-Monitor-FL kit	
12	HBV_DNA_probe	Available in AmpliSens® HBV-Monitor-FL kit	
13	cccDNA_f	CCGTGTGCACTTCGCTTCA	qRT-PCR HBV cccDNA level
14	cccDNA_r	GCACAGCTTGGAGGCTTGA	
15	cccDNA_probe	FAM-CATGGAGACCACCGTGAACGCCC-BHQ1	
16	bglobin_f	Available in V31-FEP-CE - AMPLISENS® HPV HCR-SCREEN kit	qRT-PCR reference gene
17	bglobin_r	Available in V31-FEP-CE - AMPLISENS® HPV HCR-SCREEN kit	
18	bglobin_probe	Available in V31-FEP-CE - AMPLISENS® HPV HCR-SCREEN kit	
19	DNMT3A_f	CCGGAACATTGAGGACATCT	qRT-PCR DNMT3A expression
20	DNMT3A_r	CAGCAGATGGTGCAGTAGGA	

21	APOBEC3A_f	AGATGGAGTCTGGTACTGTCG	qRT-PCR APOBEC3A expression
22	APOBEC3A_r	GAGGCAGGAGAGTAGCGT	
23	APOBEC3B_f	GACCCTTTGGTCCTTCGAC	qRT-PCR APOBEC3B expression
24	APOBEC3B_r	GCACAGCCCCAGGAGAAG	
25	MxA_f	GGTGGTCCCCAGTAATGTGG	qRT-PCR MxA expression
26	MxA_r	CGTCAAGATTCCGATGGTCCT	
27	PKR_f	GCCGCTAAACTTGCATATCTTCA	qRT-PCR PKR expression
28	PKR_r	TCACACGTAGTAGCAAAAGAACC	
29	DNA-PKcs_f	TTACATGACGCTGGCAGATTTC	qRT-PCR DNA-PKcs expression
30	DNA-PKcs_r	TTTCAACATTTTCTCCACCACAA	
31	Mre11a_f	GAAGACATTTTTCCTACCACTTCAAAG	qRT-PCR Mre11a expression
32	Mre11a_r	CTTTCGATACTTGACTCTGGGACAT	
33	Rad51_f	TCACGGTTAGAGCAGTGTG	qRT-PCR Rad51 expression
34	Rad51_r	AACAGCCTCCACAGTATGG	
35	ATM_f	AGTTTCATCTTCCGGCCTCT	qRT-PCR ATM expression
36	ATM_r	GCTGTGAGAAAACCATGGAAG	
37	ATR_f	AACATTCGTGGCATTGACTG	qRT-PCR ATR expression
38	ATR_r	AAGCAAGGTGATCTCATCCG	
39	Ultramer_U6_f	TATATAGGATCCGAGGGCCTATTTCCCATGATTCCTTCATATTTG	sgRNA-encoding PCR products preparation
40	Ultramer_SP_r	TATATAGCTAGCAAAAAAAGCACCGACTCGG	sgRNA-encoding PCR products preparation
41	sgA3A_f	GCTAATGAGGGTGGCACACTGTTTTAGAGCTAGAAATAG	sgRNA_APOBEC3A-encoding PCR product preparation
42	sgA3A_r	AGTGTGCCACCCTCATTAGCCGGTGTTTCGTCCTTTC	sgRNA_APOBEC3A-encoding PCR product preparation
43	sgA3B_f	ATTGGAGGTTCCTCTGCCAGGTTTTAGAGCTAGAAATAG	sgRNA_APOBEC3B-encoding PCR product preparation
44	sgA3B_r	CTGGCAGAGGAACCTCCAATCGGTGTTTCGTCCTTTC	sgRNA_APOBEC3B-encoding PCR product preparation

Target gene	Cycling conditions (denaturation/annealing/elongation)
GAPDH	94 °C - 30 s/54 °C - 30 s/72 °C - 30 s
pgRNA	94 °C - 15 s/62 °C - 60 s/72 °C - 10 s
S-mRNA	94 °C - 15 s/60 °C - 60 s/72 °C - 10 s
HBV DNA	94 °C - 5 s/60 °C - 20 s/72 °C - 15 s
cccDNA	94 °C - 15 s/60 °C - 60 s/72 °C - 10 s
b-globin	94 °C - 5 s/60 °C - 20 s/72 °C - 15 s
DNMT3A	94 °C - 30 s/50 °C - 30 s/72 °C - 30 s
APOBEC3A	94 °C - 15 s/51 °C - 30 s/72 °C - 5 s
APOBEC3B	94 °C - 20 s/54 °C - 15 s/72 °C - 10 s
MxA	94 °C - 30 s/54 °C - 30 s/72 °C - 20 s
PKR	94 °C - 30 s/53 °C - 30 s/72 °C - 20 s
DNA-PKcs	94 °C - 30 s/50 °C - 30 s/72 °C - 30 s
Mre11A	94 °C - 30 s/50 °C - 30 s/72 °C - 30 s
Rad51	94 °C - 30 s/50 °C - 30 s/72 °C - 30 s
ATM	94 °C - 30 s/54 °C - 30 s/72 °C - 5 s
ATR	94 °C - 30 s/56 °C - 30 s/72 °C - 7 s

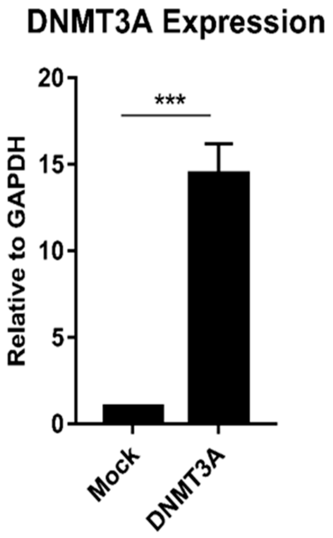

Figure S1. Relative expression of DNMT3A in Mock-treated vs DNMT3A-transfected HepG2-1.1merHBV cells. *$p < 0.05$, **$p < 0.01$, ***$p < 0.001$, ****$p < 0.0001$.

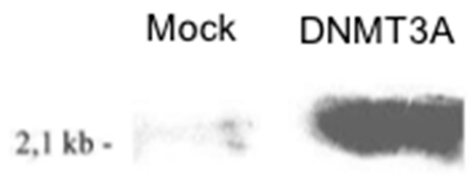

Figure S2. Southern blot analysis of HBV cccDNA from HepG2-1.1merHBV cells. Mock—HepG2-1.1merHBV cells with activated HBV expression; DNMT3A—HepG2-1.1merHBV cells with activated HBV expression and overexpression of DNTM3A.
